# Implementing a Collaborative Sepsis Protocol on the Time to Antibiotics in an Emergency Department of a Saudi Hospital: Quasi Randomized Study

**DOI:** 10.1155/2014/410430

**Published:** 2014-04-08

**Authors:** Rifat S. Rehmani, Javed I. Memon, Ayman Al-Gammal

**Affiliations:** ^1^Department of Emergency Medicine, King Abdulaziz Hospital, P.O. Box 2477, Al-Ahsa, Saudi Arabia; ^2^Intensive Care, Department of Medicine, King Abdulaziz Hospital, P.O. Box 2477, Al-Ahsa 31982, Saudi Arabia; ^3^Infectious Diseases/Department of Medicine, King Abdulaziz Hospital, Al-Ahsa 31982, Saudi Arabia

## Abstract

*Background*. The objective of this study is to evaluate the impact of an ED sepsis protocol on the time to antibiotics for emergency department (ED) patients with severe sepsis. *Methods*. Quasiexperimental prospective study was conducted at the emergency department. Consecutive patients with severe sepsis were included before and after the implementation of a sepsis protocol. The outcome measures were time from recognition of severe sepsis/septic shock to first antibiotic dose delivery and the appropriateness of initial choice of antibiotics based on the presumed source of infection. *Results*. There were 47 patients in preintervention group and 112 patients in postintervention group. Before implementation, mean time from severe sepsis recognition to delivery of antibiotics was 140 ± 97 minutes. During the intervention period, the mean time was 68 ± 67 minutes, with an overall reduction of 72 minutes. The protocol resulted in an overall improvement of 37% in the compliance, as 62% received appropriate initial antibiotics for the presumed source of infection as compared to 25% before the start of protocol. *Conclusion*. Implementation of ED sepsis protocol improved the time from recognition of severe sepsis/septic shock to first antibiotic dose delivery as well as the appropriateness of initial antibiotic therapy.

## 1. Background

Mortality from sepsis remains unacceptably high despite recent advances in diagnostic procedures, antimicrobial treatment, and supportive care [1]. While there are no prospective outcome trials to support early administration of antibiotics, prompt institution of antimicrobial therapy that is active against the causative pathogen(s) is crucial in the treatment of patients with severe sepsis [2]. In fact, the Surviving Sepsis Campaign strongly recommends initiating antibiotic therapy within the first hour of recognition of severe sepsis, after suitable cultures have been obtained [3]. It further recommends that the initial selection of antimicrobial therapy should be broad enough to cover all likely pathogens. There is ample evidence that failure to initiate appropriate therapy (i.e., therapy with activity against the pathogen that is subsequently identified as the causative agent) has adverse consequences on patient outcome [4–6]. Prior studies in severe sepsis have demonstrated improved outcomes when adequate empiric antimicrobial agents are initiated in a timely manner [7].

Despite strong evidence for the efficacy and cost-effectiveness of systematic implementation of the SSC guidelines, translation of the guidelines into clinical practice has been slow, incomplete, and delayed [8, 9]. An analysis of data from 165 hospitals of more than 15,000 patients submitted to the Surviving Sepsis Campaign demonstrated that only 68% of patients received broad-spectrum antibiotics within 3 hrs of ED presentation and found reduced hospital mortality in patients that were compliant to the recommendations on antibiotics timing [10].

Given the challenges in many EDs, administration of antimicrobial therapy within this 1 hr time frame is difficult, and several specific interventions/protocols are implemented to streamline ordering and delivery of antibiotics [11–17]. Antimicrobial treatment guidelines have been developed for effective treatment and to decrease treatment diversity, prevent treatment delay, and reduce the unnecessary use of broad-spectrum antimicrobials to avoid antimicrobial resistance. We developed an empiric antibiotic guideline based on the suspected source of infection to improve the antibiotic selection. Our hypothesis was that the use of this guideline would decrease the time to first dose antibiotic for patients with severe sepsis at our institution.

## 2. Methods

The detailed methods of the study have been described [18].

### 2.1. Design and Setting

Quasiexperimental study was designed, included before- and after-intervention groups, to measure the impact of the protocol on the mean time to antibiotics administration and the appropriateness of antibiotic selection. The study was conducted at the Emergency Department (ED) of King Abdulaziz Hospital, with 65,000 patient visits yearly.

The preprotocol period began from Jan, 2008, and concluded on Mar, 2009; the postprotocol period began from July 2009, and concluded on June 2011. Consecutive patients presented to ED with severe sepsis and septic shock was included. The intervention was introduced over a 3-month period during which no patient data were collected.

### 2.2. Eligibility

Patients aged 18 years or older, who met criteria for severe sepsis in the ED, presented with suspected infection and ≥2 SIRS criteria.

Study was approved by the Regional Scientific Committee and Institutional Review Board, with waiver from informed consent.

### 2.3. Protocol Development and Implementation

At the time of study conception, there was no written management protocol for patients with severe sepsis or septic shock in the hospital, and antibiotics were selected at the discretion of treating physician.

The ED sepsis protocol was developed by a multidisciplinary team, based on perceived need within the institution. It included physicians from the emergency department (ED), the intensive care service, the ID consultant, and pharmacy staff. During the brainstorming sessions, we found the mean time to antibiotics delivery from the time criteria for severe sepsis were met was 140 minutes along with the reasons that influenced the time to antibiotic delivery. First, our infectious disease (ID) service had exclusive prescribing privileges for certain broad-spectrum antibiotics. Before initiating any of these antibiotics, clinicians were required to take the consent of the on-call ID consultant to use the antibiotics empirically. The pharmacy also required verbal confirmation from the ID consultant before dispensing the antibiotics. There was also a substantial delay in processing the antibiotic orders and delivery through our automated pneumatic tube system. We also found that there was no real urgency among the ED staff for early initiation of antibiotics.

The protocol package consisted of an early recognition patient screening tool for sepsis and appropriate diagnostic and therapeutic interventions including initial empiric antibiotic selection guidelines according to the suspected source of sepsis, based on the institution's specific bacterial resistance patterns.  Table 1 is a summary of the empiric antibiotic guidelines during the study period. Pharmacy was engaged in the process to ensure that appropriate antibiotics were sent to the emergency department within 30 minutes. The multidisciplinary team that developed the protocol agreed that the use of recommended antibiotic therapies would substitute for the preordering ID approval.

Before implementing the protocol, an educational program in the form of lectures was also designed for the physicians and nursing and respiratory colleagues. After the implementation of protocol, antibiotics were prescribed in accordance with the recommended guidelines.

### 2.4. Data Collection and Outcome Measures

The following variables are abstracted from patients with sepsis: triage time, bed assignment time, physician assigned to patient, time when criteria for severe sepsis met, time of initiation of antibiotics, initial choice of empiric antibiotic based on source of infection, and the emergency physician's diagnosis. In addition, clinical and demographic characteristics of all patients, along with ED volume, were recorded. The visit timeline data was used to calculate the time from the criteria for severe sepsis met to first antibiotic dose delivery [CTA].

The time zero was defined as the time when severe sepsis or septic shock was recognized and sepsis resuscitation bundle initiated.

Our primary outcome was the mean time to antibiotic administration in the ED in patients meeting criteria for severe sepsis before and after the implementation of an ED sepsis protocol. Our secondary outcome was in compliance with the published Surviving Sepsis Campaign guidelines for time to antibiotics in severe sepsis and appropriateness of initial empiric antibiotic therapy in severe sepsis based on locally published guidelines. Appropriate therapy was defined as receipt of any combination of the recommended antibiotics. Partially appropriate therapy was defined as the receipt of only one of a recommended combination of antibiotics, and inappropriate therapy was defined as not having received any of the recommended antibiotics.

### 2.5. Statistical Analysis

The pilot data suggested that mean time of antibiotic administration was approximately 140 minutes. To become clinically important after the protocol implementation, we defined 50% reduction in time to antibiotics delivery. A sample size of 38 achieves 95% power to detect a difference of 50% between the null hypothesis mean of 140.0 and the alternative hypothesis mean of 70.0 with an estimated standard deviation of 97.0 and with a significance level (alpha) of 0.05 using a two-sided Wilcoxon test assuming that the actual distribution is normal.

Frequencies and means (±SD) were used to describe the sample, and differences in baseline characteristics and potential confounders between the two study groups were examined using chi-squared test and *t*-test as appropriate. All data was entered into a Microsoft Access 2000 database (Microsoft Corp., Redmond, WA). Statistical analysis was done with the Statistical Package for the Social Sciences (SPSS), version 18.0. Statistical significance was set at the 5% level, and all tests were two-tailed.

## 3. Results

### 3.1. Comparison of Intervention Group with Preintervention Group

The preintervention group included 57 cases; ten patients were excluded because of acute pulmonary edema and acute coronary syndrome. The mean age of the patients was 72 years, 51% were male and 55% had the septic shock. The major sources of infection were urinary tract (30%) and chest (28%).

The intervention group included 124 cases; 12 patients excluded because of acute pulmonary edema and acute coronary syndrome. The mean age of the patients was 66 years, 55% were male and 71% had the septic shock. The major sources of infection were again chest (36%) and urinary tract (24%).

The baseline patients' characteristics and co-morbid of the study cohort were similar (Table 2), with no statistically significant differences in age, sex, or APACHE II score. The main sources of sepsis were pneumonia and urinary tract infections in both periods.

### 3.2. Pattern of Antibiotic Coverage

The pattern of antimicrobial coverage prescribed was significantly altered by the antibiotic guidelines. Compared with the before-guideline group, patients in the after-guideline group more frequently received broad-spectrum coverage of both Gram-positive and Gram-negative pathogens (63% versus 24%, *P* = 0.01). The use of combination Gram-negative therapy also increased, although the difference was not significant (42% versus 23%; *P*, not significant).

### 3.3. Time to Antibiotics in Patients with Severe Sepsis

We observed a statistically significant decrease in time to antibiotics from the time criteria for severe sepsis were met (CTA), after implementation of the ED sepsis protocol, *P* value = 0.0001 (Table 3). The mean CTA time for the presepsis-protocol group was 140 (95% CI 102–180) minutes. The implementation of protocol led to a mean CTA time of 68 (95% CI 35–102) minutes.

Patients receiving antibiotics within 1 hour of recognition of severe sepsis as outlined by the SSC guidelines increased from 12.0% (95% CI, 9.5% to 24.0%) in preimplementation group to 52.0% (95% CI, 41.6% to 63.3%) in postimplementation group (*P* = 0.01).

### 3.4. Appropriateness of Initial Empiric Antibiotic Coverage

As shown in Table 3, appropriate initial empiric antibiotics as described in the guidelines were received in 11 (25%) patients. Partially appropriate antibiotic coverage was administered in 16 (36%) and inappropriate coverage in 17 (39%). After the implementation of protocol, appropriate initial empiric antibiotic therapy was received in 55 (62%) patients. Partially appropriate coverage was administered in 21 (24%) and inappropriate coverage in 13 (14%). The protocol, therefore, resulted in an improvement of 37% in the number of patients initially receiving appropriate antibiotics.

## 4. Discussion

Survival from severe sepsis/septic shock depends on rapid recognition, resuscitation, and treatment with appropriate antibiotic therapy. Early goal-directed therapy in the first 6 hours after presentation of severe sepsis has shown to improve mortality with a number needed to treat 6 patients to prevent 1 death [19].

Observational studies have shown a significant reduction in mortality when antibiotics are administered within the first 4 to 8 hours of hospital presentation [20, 21]. We found that our intervention to increase recognition of severe sepsis resulted in earlier administration of appropriate antibiotic therapy. Similar to earlier study [17], implementation of our ED sepsis protocol appears to have significantly decreased the time from recognition of severe sepsis to antibiotic administration. With a statistically and clinically significant improvement in time to antibiotics in severe sepsis, an ED-based protocol has the potential to markedly improve morbidity and mortality in this patient population presenting to the ED.

We found a significant improvement in the time to delivery of antibiotics. Our time to first dose was 68 minutes, 72 minutes faster than before protocol implementation. As demonstrated [22, 23], earlier delivery of antibiotics to patients with septic shock dramatically improves outcomes. The earlier studies revealed that time to effective antimicrobial therapy in septic shock had the strongest association with outcome [22], and a delay in antibiotics until after shock recognition was independently associated with mortality [13]. It is shown that in patients with severe sepsis and septic shock, the risk of in-hospital mortality was 8 times greater in patients receiving inadequate therapy within the first 24 hours compared with those receiving adequate empiric antibiotic therapy [24].

There are many reasons for the decrease in time to first dose of antibiotic. Following a written sepsis protocol increased the recognition of sepsis and septic shock by ED providers. In addition, the protocol offered more access to broad-spectrum antibiotics without the involvement of ID service. Similarly, the involvement of pharmacy services also expedites delivery of antibiotics to ED. Finally, the increased awareness by educational sessions that were carried out before the start of the protocol (the “Hawthorne effect”) could have accounted for this improvement [25].

We believe that the delays seen in initiation of antibiotics in severe sepsis and septic shock are often a product of poor early recognition. The time to antibiotic therapy can be used as a surrogate marker of early recognition and prompt management. In an effort to streamline the recognition and management of these patients, we devised a sepsis protocol that included a patient screening tool that could be activated at triage or at the bedside, as well as a data order set including recommended initial empiric antibiotics based on suspected source of infection.

Previous studies have found that an inappropriate antimicrobial agent is received by 35% of cases where the physician chooses the agent based on clinical judgment alone [26]. Our data support this finding. Patients in the before-intervention group received single antibiotic that covered only part of the bacterial spectrum, whereas patients in the after-intervention group frequently received combination therapy that covered the entire bacterial spectrum. It is found that the use of local microbiological data increases the likelihood of effective therapy [27]. Our results also revealed significant improvement in the adequacy of empiric antimicrobial therapy by the use of an institution-specific antibiotic guideline. In a previous study of similar design and similar intervention, using an antimicrobial guide on the severe sepsis order sheet resulted in improved adequacy of antibiotic therapy [28]. Despite the multidisciplinary education, the guidelines were either not followed or only partially followed in 38% of cases. We hypothesize that personal preferences and not practicing evidence based medicine could be the reasons for the poor adherence.

We also found that although the protocol had a significant improvement on the time severe sepsis criteria were met to time of antibiotic administration, the real benefit in time to antibiotics administration occurred once the sepsis was recognized early. At the start of the project, we were hoping that the protocol would be initiated at triage for the earlier identification of these patients; however, we found that it was activated only after being seen by the physicians.

There are few limitations to this study. First, it is a single center study, which may not reflect care provided at all hospitals. Furthermore, we did not design this study to detect a difference in outcome of patients treated for sepsis before and after the implementation of protocol.

## 5. Conclusions

This study demonstrates that the potential beneficial effects of the development and implementation of a sepsis protocol that emphasized on early broad-spectrum antibiotic based on the presumed source of infection can improve the time to antibiotic and the appropriateness of empiric antibiotic therapy.

Further protocol modification is in place, “to activate the sepsis protocol at the triage” to further reduce the time to first dose of antibiotic.

## Figures and Tables

**Table 1:** Empiric antibiotic therapy for cases of severe sepsis or septic shock.

Suspected source of infection	Antibiotic guidelines
Lung	
Community acquired	Moxifloxacin + cefotaxime or ceftriaxone or ceftazidime
Hospital acquired	Imipenem or meropenem or cefepime
Abdomen	
Community acquired	Imipenem or meropenem or piperacillin/tazobactam ± aminoglycoside
Hospital acquired	Imipenem or meropenem or piperacillin/tazobactam ± aminoglycoside (consider amphotericin B)
Skin and soft tissue	
Community acquired	Vancomycin + imipenem or meropenem or piperacillin/tazobactam
Hospital acquired	Vancomycin plus Cefepime or piperacillin/tazobactam
Urinary tract	
Community acquired	Ciprofloxacin or ampicillin and gentamycin
Hospital acquired	Vancomycin and cefepime or carbapenem
Meningitis	
Community acquired	Vancomycin + ceftriaxone or cefepime
Hospital acquired	Vancomycin + cefepime or ceftazidime
Neutropenic	
Community acquired	Carbapenem or cefepime or ceftazidime or piperacillin/tazobactam + aminoglycoside + Vancomycin (if having CVC catheter)
Hospital acquired	Same as above
Undetermined	
Community acquired	Vancomycin + imipenem or meropenem or cefepime
Hospital acquired	Same as above

Recommended starting antibiotic dosage for treating sepsis (normal renal function): imipenem: 0.5 gm IV; meropenem: 1.0 gm IV; piperacillin/tazobactam: 3.375 gm IV; cefepime: 1.2 gm IV; ciprofloxacin: 400 mg IV; moxifloxacin: 400 mg IV; vancomycin: 1 g IV; ceftriaxone: 2 gm IV; cefotaxime: 2 gm IV; ceftazidime: 2 g IV.

**Table 2:** Patients characteristics and clinical data.

Variables	Results		*P* value
	Preintervention group 46	Postintervention group 108	
Age	71.7 ± 18.9	66.2 ± 18.8	0.13
Sex (male)	24 (51)	61 (55)	0.31
Septic shock	26 (55)	80 (71)	0.06
Sepsis screening			
Mean arterial pressure (mm Hg)	60.4 ± 16.3	56.9 ± 9.9	0.71
Heart rate (beats/min)	109 ± 22.0	104 ± 23.7	0.09
Respiratory rate (breaths/min)	24.0 ± 6.0	24.8 ± 7.2	0.74
Temperature (°C)	36.9 ± 1.2	37.2 ± 1.2	0.66
Lactate mmol/L	3.6 ± 2.5	3.5 ± 2.5	0.66
Severity of illness			
APACHE II	20.4 ± 7.4	20.7 ± 5.6	0.29
Comorbid conditions			
Diabetes mellitus	31 (66)	72 (64)	0.72
Hypertension	33 (70)	76 (68)	0.6
Chronic kidney disease	7 (15)	16 (14)	0.8
Malignancy	6 (13)	13 (11)	0.6
Source of infection			
Urinary tract	14 (30)	27 (24)	0.36
Pneumonia	13 (28)	40 (36)	0.14
Abdomen	7 (15)	13 (12)	0.12
Soft tissue and skin	3 (7)	9 (8)	0.87
Others, with undetermined source	5 (11)	9 (8)	0.64
Mixed (>one source)	4 (9)	14 (12)	0.79

Results expressed as mean ± SD or *n* (%); APACHE: acute physiology and chronic health evaluation.

**Table 3:** Outcome measures.

Outcome variables	Results		*P* value
	Preintervention group	Postintervention group	
Primary outcome			
CTA	140 minutes	68 minutes	0.0001
Secondary outcome			
Appropriate antibiotic coverage	11 (25.0%)	67 (62.0%)	0.009
Partially appropriate antibiotic coverage	17 (37.5%)	26 (24.0%)	0.013
Complete inappropriate antibiotic coverage	17 (37.5%)	15 (14.0%)	

Results in mean or numbers (%).

CTA: time from severe sepsis/septic shock criteria met to first antibiotic dose delivery.

## References

[1] Angus DC, Linde-Zwirble WT, Lidicker J, Clermont G, Carcillo J, Pinsky MR (2001). Epidemiology of severe sepsis in the United States: analysis of incidence, outcome, and associated costs of care. *Critical Care Medicine*.

[2] Rivers EP, Katranji M, Jaehne KA (2012). Early interventions in severe sepsis and septic shock: a review of the evidence one decade later. *Minerva Anestesiologica*.

[3] Dellinger RP, Levy MM, Carlet JM (2008). Surviving sepsis campaign: international guidelines for management of severe sepsis and septic shock: 2008. *Critical Care Medicine*.

[4] Leibovici L, Shraga I, Drucker M, Konigsberger H, Samra Z, Pitlik SD (1998). The benefit of appropriate empirical antibiotic treatment in patients with bloodstream infection. *Journal of Internal Medicine*.

[5] Ibrahim EH, Sherman G, Ward S, Fraser VJ, Kollef MH (2000). The influence of inadequate antimicrobial treatment of bloodstream infections on patient outcomes in the ICU setting. *Chest*.

[6] Bochud P-Y, Bonten M, Marchetti O, Calandra T (2004). Antimicrobial therapy for patients with severe sepsis and septic shock: an evidence-based review. *Critical Care Medicine*.

[7] Varpula M, Karlsson S, Parviainen I, Ruokonen E, Pettilä V (2007). Community-acquired septic shock: early management and outcome in a nationwide study in Finland. *Acta Anaesthesiologica Scandinavica*.

[8] Carlbom DJ, Rubenfeld GD (2007). Barriers to implementing protocol-based sepsis resuscitation in the emergency department—results of a national survey. *Critical Care Medicine*.

[9] Trzeciak S, Dellinger RP, Abate NL (2006). Translating research to clinical practice: a 1-year experience with implementing early goal-directed therapy for septic shock in the emergency department. *Chest*.

[10] Levy MM, Dellinger RP, Townsend SR (2010). The surviving sepsis campaign: results of an international guideline-based performance improvement program targeting severe sepsis. *Critical Care Medicine*.

[11] Tipler PS, Pamplin J, Mysliwiec V, Anderson D, Mount CA (2013). Use of a protocolized approach to the management of sepsis can improve time to first dose of antibiotics. *Journal of Critical Care*.

[12] Hitti EA, Lewin JJ, Lopez J (2012). Improving door-to-antibiotic time in severely septic emergency department patients. *Journal of Emergency Medicine*.

[13] Puskarich MA, Trzeciak S, Shapiro NI (2011). Association between timing of antibiotic administration and mortality from septic shock in patients treated with a quantitative resuscitation protocol. *Critical Care Medicine*.

[14] Jones AE, Troyer JL, Kline JA (2011). Cost-effectiveness of an emergency department-based early sepsis resuscitation protocol. *Critical Care Medicine*.

[15] Casserly B, Baram M, Walsh P, Sucov A, Ward NS, Levy MM (2011). Implementing a collaborative protocol in a sepsis intervention program: lessons learned. *Lung*.

[16] Sweet DD, Jaswal D, Fu W (2010). Effect of an emergency department sepsis protocol on the care of septic patients admitted to the intensive care unit. *Canadian Journal of Emergency Medicine*.

[17] Francis M, Rich T, Williamson T, Peterson D (2010). Effect of an emergency department sepsis protocol on time to antibiotics in severe sepsis. *Canadian Journal of Emergency Medicine*.

[18] Memon JI, Rehmani RS, Alaithan AM (2012). Impact of 6-hour sepsis resuscitation bundle compliance on hospital mortality in a Saudi hospital. *Critical Care Research and Practice*.

[19] Rivers E, Nguyen B, Havstad S (2001). Early goal-directed therapy in the treatment of severe sepsis and septic shock. *The New England Journal of Medicine*.

[20] Houck PM, Bratzler DW, Nsa W, Ma A, Bartlett JG (2004). Timing of antibiotic administration and outcomes for medicare patients hospitalized with community-acquired pneumonia. *Archives of Internal Medicine*.

[21] Meehan TP, Fine MJ, Krumholz HM (1997). Quality of care, process and outcomes in elderly patients with pneumonia. *Journal of the American Medical Association *.

[22] Kumar A, Roberts D, Wood KE (2006). Duration of hypotension before initiation of effective antimicrobial therapy is the critical determinant of survival in human septic shock. *Critical Care Medicine*.

[23] Gaieski DF, Mikkelsen ME, Band RA (2010). Impact of time to antibiotics on survival in patients with severe sepsis or septic shock in whom early goal-directed therapy was initiated in the emergency department. *Critical Care Medicine*.

[24] Garnacho-Montero J, Garcia-Garmendia JL, Barrero-Almodovar A, Jimenez-Jimenez FJ, Perez-Paredes C, Ortiz-Leyba C (2003). Impact of adequate empirical antibiotic therapy on the outcome of patients admitted to the intensive care unit with sepsis. *Critical Care Medicine*.

[25] Festinger L, Katz D (1953). *Research Methods in Behavioral Sciences*.

[26] Vogelaers D, de Bels D, Forêt F (2010). Patterns of antimicrobial therapy in severe nosocomial infections: empiric choices, proportion of appropriate therapy, and adaptation rates-a multicentre, observational survey in critically ill patients. *International Journal of Antimicrobial Agents*.

[27] Lancaster JW, Lawrence KR, Fong JJ (2008). Impact of an institution-specific hospital-acquired pneumonia protocol on the appropriateness of antibiotic therapy and patient outcomes. *Pharmacotherapy*.

[28] Hanzelka KM, Pierce CA, Beardsley JR, Williamson JC, Morris PE (2010). Influence of an institution-specific sepsis protocol on the adequacy of empiric antimicrobial therapy. *Hospital Pharmacy*.

